# Hemangioendothelioma as a rare cause of lower gastrointestinal bleeding - a case report

**DOI:** 10.1016/j.ijscr.2024.110381

**Published:** 2024-09-30

**Authors:** Seyed Reza Fatemi, Alireza Zahedi, Mina Moghtaderi, Mohammad Reza Hashempour, Zhaleh Mohsenifar, Seyed Ali Fatemi

**Affiliations:** a Gastroenterology and Liver Diseases Research Center, Shahid Beheshti University of Medical Sciences, Tehran, Iran; b Colorectal Division of Surgical Ward, Taleghani Hospital, Shahid Beheshti University of Medical Sciences, Tehran, Iran; c Faculty of Medicine, Shahid Beheshti University of Medical Sciences, Tehran, Iran; d University of Toronto, Toronto, Canada

**Keywords:** Gastrointestinal hemorrhage, Hemangioendothelioma, Intestinal neoplasms, Endoscopy, Laparotomy

## Abstract

**Introduction and importance:**

Gastrointestinal bleeding is a serious health threat, especially when it originates from the small intestine, often termed small bowel bleeding (SBB). Hemangioendothelioma, a rare vascular tumor, can be a significant yet uncommon cause of gastrointestinal bleeding. This case report highlights hemangioendothelioma's diagnostic challenges and clinical significance in SBB.

**Case presentation:**

A 16-year-old female experienced six months of intermittent massive rectorrhagia and melena, along with nausea, vomiting, loss of appetite, and abdominal pain. Initial endoscopic investigations, including colonoscopy and enteroscopy, did not identify the bleeding source. Imaging studies, including a CT scan and Meckel's scintigraphy, were also inconclusive. Persistent bleeding and a significant drop in hemoglobin levels led to exploratory laparotomy with intraoperative enteroscopy, which revealed a nodular lesion in the small intestine. Histopathological examination confirmed the lesion as hemangioendothelioma. Surgical resection of the lesion stopped the bleeding, and the patient recovered without complications, remaining asymptomatic during follow-ups at one and six months post-surgery.

**Clinical discussion:**

Hemangioendothelioma is a rare, locally aggressive vascular tumor that can present with abdominal pain, bowel obstruction, anemia, and gastrointestinal bleeding. Due to its rarity and location, it often goes undetected in conventional endoscopy. The definitive diagnosis is made through histopathological examination, which shows characteristic endothelial cells and vascular structures. Surgical excision is the primary treatment, although there is a risk of recurrence and metastasis.

**Conclusion:**

This case underscores hemangioendothelioma's diagnostic challenges and clinical relevance in obscure gastrointestinal bleeding. Intraoperative enteroscopy plays a crucial role in the diagnosis and management. Clinicians should consider hemangioendothelioma in similar cases to ensure appropriate treatment and management.

## Highlights

• A 16-year-old female presented with intermittent massive rectorrhagia and melena, ultimately diagnosed with hemangioendothelioma.
• Extensive endoscopic and imaging investigations initially failed to detect the bleeding source.
• A small nodular lesion was found intraoperatively and confirmed to be hemangioendothelioma via histopathological examination.
• Surgical resection of the lesion successfully stopped the bleeding, and the patient recovered without complications.
• This case highlights the diagnostic challenges of obscure gastrointestinal bleeding and the rarity of hemangioendothelioma as a cause.

## 1 Introduction

Gastrointestinal bleeding is one of the potentially life-threatening events that can manifest in several forms and can be classified as overt, occult, or obscure [1]. Small bowel bleeding (SBB) refers to bleeding of unknown origin that persists or recurs after initial negative results from endoscopy and colonoscopy [2], Typically, this type of bleeding originates from the small intestine [1]. Vascular, inflammatory, iatrogenic, tumor and diverticular disorders play a role in the etiology of SBB. Bleeding tumors include gastrointestinal stromal tumors, adenocarcinoma, metastatic tumors, malignant lymphoma, Putz-Jeggers polyp, inflammatory fibroid polyp, ectopic pancreas, and lipoma [3]. Hemangioendothelioma is a rare but notable cause of GI bleeding. In this case, we present a patient with SBB, and based on the pathology report, the specimen resected from the small intestinal wall was found to be compatible with hemangioendothelioma.

## 2 Case presentation

A 16-year-old female was admitted to the emergency ward of a hospital with complaints of intermittent massive rectorrhagia and melena in the last 180 days ago. She also reported experiencing nausea, vomiting, loss of appetite, and abdominal pain. The patient had no significant personal or familial underlying medical condition. She had no history of bleeding diseases, but she had abdominal pain in periumbilical site occur generally. The patient reported no past surgical history. The patient did not report any use of medications. The patient declined smoking and alcohol consumption.

A physical examination revealed mild generalized tenderness throughout the abdomen, and bright blood on rectal examination. The patient was a candidate for endoscopy. The findings were normal. Then, he underwent a colonoscopy, and melena was seen in the rectum that scope could not pass. Anterograde and retrograde single balloon enteroscopy procedures were performed to continue investigations extending up to the proximal segments of the ileum. The results of these procedures were unremarkable, with no active bleeding identified. Subsequently, an ileocolonoscopy was conducted, revealing melena throughout the colon from the rectum to the cecum, as well as in the terminal ileum and throughout the distal 50 cm of the ileum. However, no specific source of bleeding was detected during the examination.

Following these endoscopic investigations, a computed tomography (CT) scan with a Visipaque (iodixanol) contrast of the abdomen and pelvis was performed. The scan revealed a hypodense subcapsular area in the fourth segment of the liver, measuring 2 mm in diameter, suggestive of focal fat infiltration. No evident signs of extravasation or contrast blush within the GI tract were observed in the CT scan. During two weeks of hospitalization and supportive measures and receiving 8 units pack cell, the patient frequently experienced melena and intermittent rectorrhagia with a period without bleeding.

Further evaluation included Meckel's scintigraphy using TC-99 m pertechnetate, which did not reveal any abnormal collections of activity during the study's dynamic and delayed phases within the abdomen and pelvis. Additionally, the scan produced negative results for the presence of a Meckel diverticulum containing gastric mucosa. Lastly, a red blood cell (RBC) scan for GI bleeding during angiographic and equilibrium phases showed no abnormal radioactivity collection during early and late imaging. Further, due to the sudden drop of hemoglobin to 4 g/dl and severe rectorrhagia, the patient became a candidate for exploratory laparotomy with intraoperative enteroscopy.

**Fig. 1:**
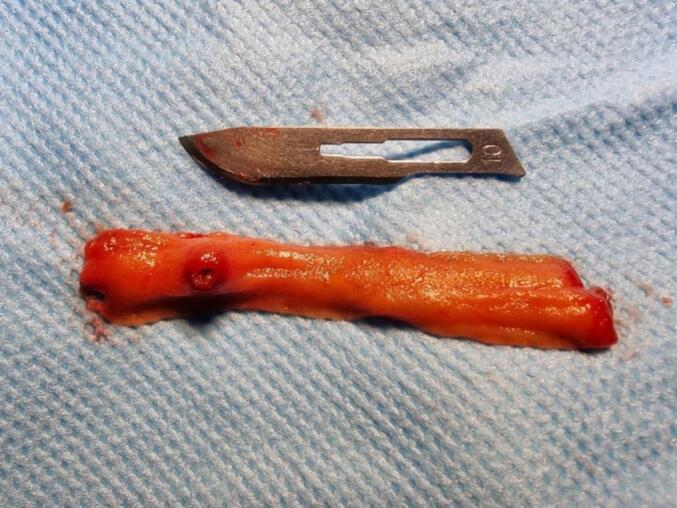
The nodular lesion in the antimesenteric wall.

**Fig. 2:**
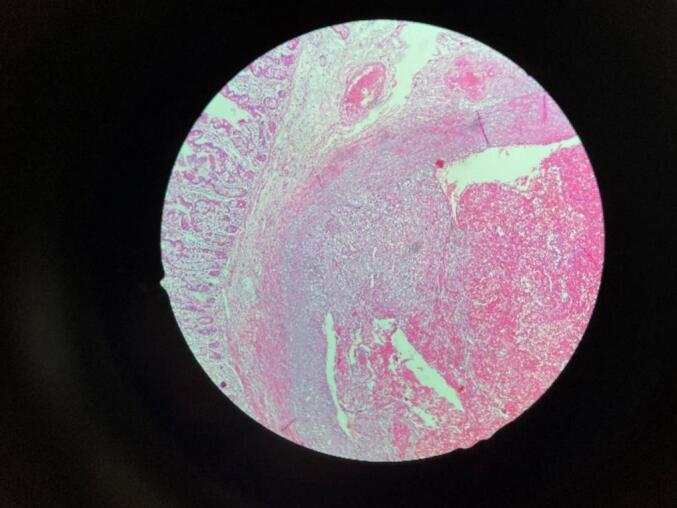
Microscopic view of the lesion.

Finally, a diagnostic laparoscopy was performed with trocar placement above the navel. There was no evidence of lesions in the stomach, small intestine and colon. By extending the incision, the small intestine was taken out from the abdomen and examined from the ileocecal valve to the ligament of Tretz. A small nodular lesion was palpated in the antimesenteric wall at 200 cm from the tritium and 200 cm from the ileocele valve. After preparation, intraoperative enteroscopy was performed from the end of the ileum to the forward, which revealed 100 cm from the terminal ileum one ulcerative lesion was seen that it had bleeding jet from around it. e. Segmental resection of the lesion was performed from the same intraoperative proximal and distal point to the lesion **(**Fig. 1**).** Segmental resection and primary repair were done with end-to-end anastomosis. No secondary lesions were seen. Enterography was performed, and the patient was transferred to the recovery department. After 48 h without bleeding and without a drop in hemoglobin, she was discharged with stable conditions and PO tolerance. The lesion was sent to pathology. Following up, the pathology report showed an enteric wall with an intraluminal proliferative endothelial lesion of the submucosal artery. Also, hemorrhage and clot were seen on it (Fig. 2). The patient was followed for one and 6 months and had no specific complaints.

Notably, this study has been reported in line with the SCARE criteria [4].

## 3 Discussion

In this study, we present a rare case of hemangioendothelioma, which presents with overt SBB presentation in a 16-year-old girl. Neoplasms in the small intestine are rare, primarily consisting of adenocarcinomas, malignant carcinoids, and leiomyosarcomas [5,6]. Hemangioendotheliomas are exceedingly rare and locally aggressive vascular tumors with borderline behavior, bridging characteristics between hemangiomas to angiosarcomas and more commonly develop in children within the first year of life or in the elderly [[7], [8], [9]]. Based on our review of previous studies, only seven cases of hemangioendothelioma have been reported so far [6,[10], [11], [12], [13], [14], [15]]. Hemangioendothelioma are categorized into four classes: epithelioid, fusiform, kaposiform, and endovascular papillary angioendothelioma, with the first two being more prevalent [9].

Hemangioendothelioma usually manifest in the skin and subcutaneous tissue of extremities and trunk, bones, mediastinum, retroperitoneum, spleen, liver, and rarely intestines [7,14]. Several risk factors have been proposed to be associated with the risk of developing hemangioendothelioma, including the use of oral contraception, inhalation of vinyl chloride, and previous exposure to radiotherapy, all contributing to the dysregulation of angiogenesis and lymphangiogenesis as the underlying mechanism [7,9].

Hemangioendothelioma has been implicated as a cause of upper gastrointestinal bleeding [16]. Clinical manifestations range from abdominal pain (14 %), perforation (21 %), mechanical and functional bowel obstruction (29 %) to anemia (43 %) and overt and occult gastrointestinal bleeding (43 %) [5]. However, in many cases, hemangioendotheliomas are not detected in conventional endoscopy due to their location in the small intestine. A life-threatening condition in patients with hemangioendothelioma is the Kassabach-Merritt syndrome, which presents with thrombocytopenia and consumption coagulopathy [17], and its early diagnosis and management impose significant importance.

The gold standard for diagnosis is the histopathological examination of the excised specimen. This examination demonstrates the presence of irregular tumor nodules containing spindle-shaped endothelial cells with ample eosinophilic cytoplasm. These cells create crescent-shaped vascular spaces, and fragmented red blood cells are present. Importantly, there is an absence of significant atypia, necrosis, and mitosis in the analyzed samples [7,18].

Some therapeutic options have been proposed for the management of the disease, and the most important is surgery. Yoshida et al. (1999) and Yasuda et al. (1989) emphasized surgery as the primary treatment for malignant hemangioendothelioma in the small intestine. They highlighted the importance of early detection and timely intervention to improve patient outcomes [6,14]. Botsford et al. (1962) reviewed one case of small intestinal tumors, including malignant hemangioendothelioma among 114 cases with other tumors, and stressed the rarity of this tumor and the need for early diagnosis to minimize complications and enhance surgical results [10]. Nakamura et al. (2017) reported a case of epithelioid hemangioendothelioma with metastasis to the mesentery, underscoring the significance of metastasis in rare cases and the need for aggressive management [11]. Odgaard et al. (2012) and Salman et al. (2018) documented cases of kaposiform hemangioendothelioma in children leading to bowel obstruction. These studies confirmed that surgical intervention is crucial for preventing severe complications [12,13]. Youn et al. (2018) also reviewed a case of kaposiform hemangioendothelioma causing bowel obstruction, reinforcing the rarity of these tumors and the necessity for surgery [15]. The review of reported cases in the literature shows that hemangioendothelioma in the small intestine is rare. Still, when it occurs, it is commonly associated with bowel obstruction or gastrointestinal bleeding. Surgery remains the primary treatment for removing these tumors, and in cases of obstruction or severe bleeding, prompt intervention is crucial. Most cases are cured with excision of the tumor; however, the tumor's recurrence and metastasis have been reported in the literature, and a 15 % mortality rate was reported as well [5]. In cases where metastasis or aggressive tumor behavior is observed, additional treatments such as laser therapy, radiation, embolization, and chemotherapy [17].

In conclusion, despite the limitation of the lack of immunohistochemistry (IHC) evaluation, our study is important due to its rarity. In this study, overt SBB was attributable to hemangioendothelioma in the small intestine, controlled with exploratory laparotomy, intraoperative enteroscopy, and resection of the lesion.

## Parental consent

Due to the presented patient being 16 years old, written informed consent was obtained from the patient's parents and guardian for publication and any accompanying images. A copy of the written consent is available for review by the Editor-in-Chief of this journal on request.

## Ethical approval

According to the guidelines of the ethics committee of the Shahid Beheshti Medical University (SBMU), case reports do not require formal ethical approval. Also, we obtain informed consent from the patients before proceeding with the case report. This is a well-established practice in the institution, as case reports are considered an important part of the medical literature and a valuable tool for sharing clinical insights and experiences with the broader medical community.

## Funding

No funding

## Author contribution

Seye Reza Fatemi and Mohammad Reza Hashempour and Zhaleh Mohsenifar and Alireza Zahedi data collection

Alireza Zahedi and Mina Moghtaderi and manuscript draft

Seyed Ali Fatemi and Seyed Reza Fatemi critical revision

## Guarantor

Dr Alireza Zahedi

Email: a.zahedi2013@gmail.com

## Conflict of interest statement

The authors have no relevant financial or nonfinancial interests to disclose.
